# Electrophysiological Correlates of Reward Anticipation in Subjects with Schizophrenia: An ERP Microstate Study

**DOI:** 10.1007/s10548-023-00984-7

**Published:** 2023-07-05

**Authors:** A. Perrottelli, G. M. Giordano, T. Koenig, E. Caporusso, L. Giuliani, P. Pezzella, P. Bucci, A. Mucci, S. Galderisi

**Affiliations:** 1 University of Campania “Luigi Vanvitelli”, Naples, Italy; 2 Translational Research Center, University Hospital of Psychiatry and Psychotherapy, University of Bern, Bern, Switzerland

**Keywords:** Schizophrenia, Anhedonia, Reward anticipation, EEG, ERPs, Brain electrical microstates

## Abstract

The current study aimed to investigate alterations of event-related potentials (ERPs) microstate during reward anticipation in subjects with schizophrenia (SCZ), and their association with hedonic experience and negative symptoms. EEG data were recorded in thirty SCZ and twenty-three healthy controls (HC) during the monetary incentive delay task in which reward, loss and neutral cues were presented. Microstate analysis and standardized low-resolution electromagnetic tomography (sLORETA) were applied to EEG data. Furthermore, analyses correlating a topographic index (the ERPs score), calculated to quantify brain activation in relationship to the microstate maps, and scales assessing hedonic experience and negative symptoms were performed. Alterations in the first (125.0–187.5 ms) and second (261.7–414.1 ms) anticipatory cue-related microstate classes were observed. In SCZ, reward cues were associated to shorter duration and earlier offset of the first microstate class as compared to the neutral condition. In the second microstate class, the area under the curve was smaller for both reward and loss anticipation cues in SCZ as compared to HC. Furthermore, significant correlations between ERPs scores and the anticipation of pleasure scores were detected, while no significant association was found with negative symptoms. sLORETA analysis showed that hypo-activation of the cingulate cortex, insula, orbitofrontal and parietal cortex was detected in SCZ as compared to HC. Abnormalities in ERPs could be traced already during the early stages of reward processing and were associated with the anticipation of pleasure, suggesting that these dysfunctions might impair effective evaluation of incoming pleasant experiences. Negative symptoms and anhedonia are partially independent results.

**Supplementary Information:**

The online version contains supplementary material available at 10.1007/s10548-023-00984-7.

## Introduction

Negative symptoms constitute a critical unmet need in the treatment of schizophrenia due to their effects on the quality of life of patients and their low responsiveness to the available pharmacological treatments (Maj et al. 2021; Galderisi et al. 2020, 2021a; Correll and Schooler 2020; Bucci et al. 2020; Fusar-Poli et al. 2022; Correll et al. 2022). These symptoms are a very heterogeneous and complex phenomenon, thus conceptualizing them has long been a contentious issue (Galderisi et al. 2013, 2016; Giordano et al. 2022b; McCutcheon et al. 2022). Currently, this construct includes five individual symptoms: anhedonia, avolition, asociality, blunted affect and alogia (Kirkpatrick et al. 2006; Galderisi et al. 2021b; Giordano et al. 2022a; Mucci et al. 2019b). Furthermore, most studies supported a two-domain model clustering of negative symptoms, named as the motivational deficit domain (consisting of anhedonia, avolition and asociality) and the expressive deficit domain (consisting of blunted affect and alogia) (Peralta et al. 2021; Blanchard and Cohen 2006; Giordano et al. 2022a; Galderisi et al. 2021b).

Anhedonia, which is believed to be a key component of the negative symptomatology (Berridge and Kringelbach 2015; Liang et al. 2022), is defined as the reduction or complete loss of the ability to experience (consummatory anhedonia) or anticipate pleasure (anticipatory anhedonia) (Liang et al. 2022; Berridge and Kringelbach 2008; Malla et al. 2022). The former reflects the ability to experience pleasure when directly engaged in enjoyable activities (Gard et al. 2007). The latter is associated with a deficit in the anticipation of pleasant experiences, which would also lead to impairments in goal-directed behavior due to a reduction in the expectation of enjoyment from future activities (Gard et al. 2007). Previous studies have shown that subjects with schizophrenia seem to be mainly impaired in the anticipation of pleasure, while no remarkable impairments have been detected in the capacity to experience pleasure in the moment (Kring and Barch 2014; Kring and Elis 2013; Gard et al. 2007).

It is believed that the clinical phenotype of anhedonia reflects the presence of dysfunctions in the cerebral networks involved in reward processing (Der-Avakian and Markou 2012; Liang et al. 2022; Zhang et al. 2016). This involves the capacity to dynamically incorporate both positive and negative feedback from the environment with the aim of increasing rewards and reducing losses over time (Saperia et al. 2019). These aspects have been studied in humans using different paradigms, amongst which the most influential one is the Monetary Incentive Delay (MID) task (Knutson et al. 2000), which focuses on the effects of reward anticipation processing. The use of functional magnetic resonance imaging (fMRI) has established the main brain regions linked to reward processing and anhedonia. Several studies have recorded fMRI during the MID task in subjects with schizophrenia (Zeng et al. 2022). These studies revealed a prominent role in the reward processing for the following brain regions: prefrontal cortex (PFC), anterior cingulate cortex (ACC), ventral striatum, insula, ventral tegmental area (VTA), substantia nigra (SN), orbital prefrontal cortex (OFC) and amygdala (Berridge and Kringelbach 2015; Der-Avakian and Markou 2012; Liang et al. 2022; Oldham et al. 2018; Knutson et al. 2001; Zeng et al. 2022; Giordano et al. 2018b; Wilson et al. 2018; Klingberg et al. 2022). Dysfunctions in these brain areas in terms of functional activation or connectivity have often been linked to impairments in reward processing or to the severity of anhedonia or negative symptoms (Waltz et al. 2018; Lambert et al. 2018; Segarra et al. 2016; Harvey et al. 2010; Fox and Lobo 2019; Arrondo et al. 2015). However, only rarely the studies characterized primary negative symptoms (Mucci et al. 2015; Giordano et al. 2021c) or investigated specifically whether anhedonia had the same correlates of other negative symptoms (Simon et al. 2010; Giordano et al. 2021c); furthermore, measures of altered functional connectivity within the cortico-striatal circuits showed an association with self-rated but not with clinician-rated scores of anhedonia and avolition (Brakowski et al. 2022).

In addition to fMRI, electroencephalography (EEG) recordings and the analysis of event-related potentials (ERPs) constitute precious tools to characterize the different neuronal steps associated to reward due to their great temporal resolution (Phillips et al. 2009; Qiu et al. 2014; Perrottelli et al. 2022). Previous studies showed that subjects with schizophrenia present alterations in ERPs related to reward anticipation as showed by differences between patients and healthy controls in the elicitation of N200 and P300, flagging the presence of aberration in the neuronal substrates sustaining evaluation of future events (Vignapiano et al. 2016, 2018; Catalano et al. 2022). In addition, alterations in reward processing, as demonstrated by abnormalities in ERPs, such as the sustained posterior negativity (SPN) (Wynn et al. 2010) or the P300 (Vignapiano et al. 2016) in subjects with schizophrenia, was found to correlate with trait anhedonia (Vignapiano et al. 2016; Wynn et al. 2010).

However, the data regarding neurophysiological abnormalities subtending reward processing impairments and anhedonia is too scarce to draw robust conclusions. Furthermore, previous EEG studies focused a-priori on specific and late-occurring ERPs, thereby failing to shed light on the early stages of reward processing.

In this frame, microstate analysis might be a useful tool for investigating early EEG components. Indeed, microstate analysis segments the continuous EEG signal into a series of quasi-stable topographies, each representing a specific step of information processing subtended by the activation of distinct neural networks. Such analyses allow analyzing the signal in a data-driven, reference-independent way, without a priori assumptions regarding generators or preferable electrode positions (Koenig et al. 2014). Furthermore, compared to some previous studies of EEG and MID task that considered specific ERPs and time windows for the analysis, microstate analysis allows the detection of electrophysiological differences without selecting a priori time windows, including early and late stages of reward anticipation.

Only one study carried out by our research group used a microstate analysis to investigate neurophysiological correlates of negative symptoms in subjects with schizophrenia (Giordano et al. 2018a). However, this study focused on data recorded with subjects at rest and not with ERPs data recorded while subjects performed a task. One of the main results of the study was that the microstate A, which seems to be related to the cerebral networks controlling visual processing of external stimuli and arousal (Tarailis et al. 2023), correlated with the Motivational Deficit domain in subjects with schizophrenia, suggesting that this domain is related to alterations in sensory processing even in the absence of a task. Interestingly, only the anticipatory anhedonia, and not the consummatory one, showed a correlation with microstate A, as the motivational deficit domain, supporting the hypothesis that deficits in the anticipation of pleasure might be underpinned by a separate neurobiological pathway than the one at the core of consummatory anhedonia (Giordano et al. 2018a).

Therefore, to broaden our knowledge of the neurophysiological correlates of reward processing and anhedonia, our study had the following main objectives: (1) to detect abnormalities in anticipation of reward during MID task using ERPs microstate analysis, without a priori assumptions on the time windows or electrodes to consider; (2) to investigate possible associations of electrophysiological markers of anticipation of reward and avoidance of loss during the MID task with hedonic experience and negative symptoms; (3) to investigate topographic differences in ERPs between subjects with schizophrenia and healthy controls and to source-localize them through standardized low resolution electromagnetic tomography (sLORETA) analysis.

## Materials and Methods

### Participants

Thirty-five subjects with a diagnosis of schizophrenia (SCZ) recruited from outpatient units of the University Psychiatric Department of Naples and twenty-six healthy controls (HC) were enrolled in the present study (Vignapiano et al. 2016).

Inclusion criteria were: (1) a clinical diagnosis of schizophrenia confirmed using the Mini International Neuropsychiatric Interview-Plus (MINI-Plus); (2) age between 18 and 65 years; (3) clinically stable (i.e., no hospitalization or change in psychotropic medication for 3 months prior to recording), in order to reduce the presence of severe positive symptoms which might lead to secondary negative symptoms; (4) treatment with second-generation antipsychotics only (Leichsenring et al. 2022); (5) a negative neurological examination; (6) a negative history of moderate intellectual disability, neurological illness, head injury with loss of consciousness, alcoholism or drug abuse or dependence in the last 6 months (except for smoking); and (7) no previous insulin coma, leucotomy or electroconvulsive therapy.

Healthy controls (HC), matched with subjects with schizophrenia (SCZ) for age (± 3 years), gender and handedness, were recruited from the community and screened with a phone interview. They were excluded if they had a past or a current DSM-IV Axis I disorder based on MINI-Plus interview or a family history of affective or psychotic disorders. Additionally, exclusion criteria included:

(1) major medical illnesses; (2) history of seizures, head injury resulting in a loss of consciousness, neurological illness, intellectual disability; (3) lifetime history of substance abuse or addiction (except for smoking) and the use of drugs which might affect central nervous system functions (e.g., hormones).

The assessment of the handedness was carried out by the Edinburgh inventory (Oldfield 1971). All subjects had normal or corrected to normal vision. A neurophysiological evaluation was carried out in all participating subjects. The Ethics Committee of the Medical Hospital of the Second University of Naples approved the study, and all subjects provided a written informed consent, after a complete description of the study.

### Psychopathological Assessment

In the whole sample anticipation and experience of pleasure were assessed trough the Temporal Experience of Pleasure Scale (TEPS) (Gard et al. 2006), which includes two subscales: anticipatory pleasure and consummatory pleasure. The 10-item anticipatory pleasure subscale is related to reward responsiveness and imagery, while the 8-item consummatory pleasure subscale is related to openness to different experiences, and appreciation of positive stimuli.

For the assessment of trait anhedonia, all participants were administered the Physical Anhedonia Scale (PAS) and the Chapman Social Anhedonia Scale (SAS) (Chapman et al. 1976). These true–false self-report measures provide indices of the pleasure derived from physical and social–interpersonal sources, respectively. PAS assessed deficits in the ability to experience pleasure from typical physical stimuli. It includes 61 items concerning the experience of pleasure related to taste, sight, touch, smell, and sex; high scores indicate severe physical anhedonia. SAS evaluated deficits in the ability to experience pleasure from non-physical stimuli such as other people, talking or exchanging expressions of feelings. It contains 40 items, and a high score indicates more severe social anhedonia.

General cognitive abilities were assessed using the Wechsler Adult Intelligence Scale-Revised (WAIS-R) in the whole experimental sample.

Measures of real-life motivation were derived from the Quality of Life Scale (QLS) (Heinrichs et al. 1984) following Nakagami and colleagues (Nakagami et al. 2010) by averaging motivation (“ability to sustain goal directed activities”), curiosity (“degree to which one is interested in his/her surroundings”), and sense of purpose (“realistic integrated life goals”) items, with higher scores indicate greater motivation.

In the patient’s group the positive and negative syndrome scale (PANSS) was used to assess positive, negative and disorganization dimensions. In particular, the positive dimension was calculated according to Wallwork and colleagues (Wallwork et al. 2012) by summing the scores on the items “delusions” (P1), “hallucinatory behavior” (P3), “grandiosity” (P5), and “unusual thought” (G9); negative dimension was assessed by summing the scores on the items “blunted affect” (N1), “emotional withdrawal” (N2), “poor rapport” (N3), “passive/apathetic social withdrawal” (N4), and “lack of spontaneity and flow of conversation” (N6), and disorganization was calculated using other three items of the PANSS scale: “conceptual disorganization” (P2), “difficulty in abstract thinking” (N5), and “poor attention” (G11).

Patients were also administered the Schedule for the Deficit Syndrome (SDS) (Kirkpatrick et al. 1989), to evaluate negative symptoms domains. The motivational deficit domain was assessed by summing the scores on the items curbing of interests, diminished sense of purpose, and diminished social drive. The expressive deficit was calculated by summing the scores on the items restricted affect, diminished emotional range, and poverty of speech (Galderisi et al. 2013, 2021b).

### Experimental Design

The electrophysiological component of the study used a modified version of the monetary incentive delay (MID) task (Vignapiano et al. 2016) (Fig. S1). In this task, the subjects must press a button within a predefined time window to win or avoid losing money. There were 4 incentive conditions—large reward, small reward, large loss, and small loss—and a neutral condition, presented in random order. The proportion of trials for the incentive conditions (18 trials each) and for the neutral one (24 trials) was the same as in the original MID task. After cue presentation (250 ms), subjects waited for a variable interval (delay; 2000–2500 ms) and then had to respond to a white target square that appeared for a variable length of time by pressing a button with the index finger of their dominant hand. After the target presentation, a feedback appeared (1650 ms), notifying subjects whether they won or did not win money on reward trials or whether they lost or did not lose money on the loss trials (Fig. S1). A total of 96 trials, each with a duration of 6 s, were completed with a total task duration of 9.6 min.

Task difficulty, related to the duration of target exposure, was based on reaction times collected during a previous 48 trial practice session. During the practice session, subjects were instructed to press the button as fast as possible irrespective of the cue type, and were excluded from analysis if they achieved less than 60% of correct responses. Subjects were also informed about the amount of money they could earn when the task was successfully performed, and after the EEG acquisition, participants were paid the amount of money they won. For both tasks, stimuli presentations and recording of reaction times were performed using the software presentation (Neurobehavioral Systems, Inc).

### EEG Data Acquisition and Preprocessing

EEG was recorded with a 32-channel digital EEG system EASYS2 (Brainscope, Prague) using a cap electrode system with 30 unipolar leads (Fpz, Fz, Cz, CPz, Pz, Oz, F3, F4, C3, C4, FC5, FC6, P3, P4, CP5, CP6, O1, O2, Fp1, Fp2, F7, F8, T3, T4,T5, T6, AF3, AF4, PO7, PO8), placed according to the 10–20 system (American Electroencephalographic Society). All the leads were referenced to the linked earlobes (a resistor of 10 kohm was interposed between the earlobe leads). A ground electrode was placed on the forehead. A horizontal electro-oculogram (hEOG) was recorded from the epicanthus of each eye, and a vertical EOG (vEOG) from the leads beneath and above the right eye for artifact monitoring. All impedances of the leads were kept at less than 5 kohm. The EEG data were filtered with a band-pass of 0.1–70 Hz and recorded with a sampling rate of 256 Hz. A calibration was performed for all channels, using a 50 µV sine wave, before each recording session. Subjects were instructed to relax, maintain a constant level of attention throughout the whole session, and avoid movements during the recording. Smokers were allowed to smoke prior to EEG recording (the last cigarette approximately 60 min before the session) to avoid the potential effects of nicotine withdrawal. All subjects were instructed to abstain from coffee and tea for at least 12 h overnight before the next morning’s experiment and to consume a light breakfast. EEG was recorded at about 9.00 am in all subjects. EEG data were analyzed using Brain Vision Analyzer software 2.0 (Brain Vision, Germany). The EEG was digitally filtered offline with a 1–15 Hz band-pass. The eye movements were corrected using independent component analysis. Independent components corresponding to artefactual sources and brain activity were separated with a manual procedure. The EEG data were manually screened for residual artifacts and recomputed to average reference. Next individual condition-wise average ERPs were computed from the artifact-free data, using a time-window from − 500 ms to + 1000 ms in reference to the cue onset times. For the “reward condition”, data recorded after the presentation of cues for small and large rewards were combined. Similarly, for the “loss condition”, the data obtained upon presentation of cues flagging the incoming possibility of obtaining small or large loss cues were combined. Based on these ERPs, we determined a suited analysis time window for the subsequent ERP microstate analyses using time-point-wise topographic consistency tests (Koenig and Melie-García 2010). Periods at the beginning and end of the where this test failed to be significant (i.e., where there was a lack of evidence of topographic consistency across subjects) were excluded from the microstate analysis.

### Microstate Analysis

Microstate analysis segments the ERP signal data-driven and reference-independent into sequences with quasi-stable map topographies, using recordings from all the electrodes. These microstates stand for distinct steps in stimulus processing and assumingly represent the activation of underlying networks (Koenig et al. 2014; Lehmann and Skrandies 1980).

Microstate analyses were conducted with Ragu (Koenig et al. 2011), where a k-means clustering algorithm was applied to the concatenated grand-means of reward, loss, and neutral conditions of the cue-related time frame and ERPs of patients and controls to identify prototypical microstate class maps. The optimal number of microstate classes was initially identified with a cross-validation procedure (Koenig et al. 2014), but later reduced to a solution with fewer classes because this solution represented the earlier stages of the stimulus processing that we were primarily interested in equally well. The thereby obtained prototypical microstate class maps were then assigned to the grand-mean of ERPs of the three conditions of patients and controls each, and the amount of variance of the ERP that was explained by the assigned microstate classes was computed as a function of time, group, and condition.

To statistically test whether the factors groups (SCZ/HC), conditions (Reward/Loss/Neutral), or their interaction affected the microstate sequence, effects observed in the actual data were compared to effects obtained with data wherein the assignment of an ERP to a certain level of the factor group and/or condition was randomized (i.e., effects observed under the null hypothesis; 5000 randomizations) (Koenig et al. 2014). A p-value of 0.05 indicates that only 5% of all effects obtained in the 5000 randomization runs were larger than the effects obtained in our real data. Six effect parameters were extracted per microstate, factor, and condition and statistically analyzed with Ragu software: (1) onset of each microstate; (2) offset of each microstate; (3) duration of each microstate, which is an index of time spent in a particular processing step; (4) sum of the variance explained (area under the curve), which is an index of the overall amount of activated brain resources associated with a particular microstate class; (5) center of gravity of time course of explained variance, which is a robust index of the distribution of a microstate class over time and (6) mean Global Field Power (GFP) (Lehmann and Skrandies 1980), which indicates overall mean signal strength (and thus the amount of simultaneously recruited neural resources by a particular processing step). For each microstate class of interest, we initially tested the full 2 group x 3 condition interaction for significance. If a microstate class occurred more than once throughout the time frame considered, the analysis was conducted separately for each of its appearances, limiting the time windows of analysis to periods of time where the given microstate class appeared only once. For microstate classes where we found significant interactions between these two factors, follow-up contrast analyses were conducted to further clarify the source of the effects. Furthermore, to be able to correlate our findings with the psychopathological assessment, we computed an individual ERP-score of the given contrast. For this purpose, the corresponding individual ERP maps were averaged within the time period of the microstates that produced a significant group × condition interaction (mean onset and offsets calculated across groups and conditions), and a mean group difference map for the condition of interest was computed, yielding what was called a template map. The individual mean maps were then projected onto this template map by computing their dot-product. This yielded, for each subject, a single ERP-score. Mathematically, this score represents, for each individual, the relative activation of those brain regions that differed between groups. Finally, using the same time windows and conditions that were significant and particularly relevant to address the given research question, the following source analyses were conducted to further clarify the functional significance of the group differences obtained.

### Source Analysis

Using the time windows of microstates that yielded a significant group × condition interaction in microstate parameters indicating intensity difference (i.e., mean GFP and area under the curve), the sources of these group differences in a given condition were modeled in the identified time period with the sLORETA inverse solution in the implementation retrieved from https://www.uzh.ch/keyinst/loreta.htm (Pascual-Marqui 2002), and using voxel-wise two-tailed *t* tests (threshold for t = 2.14, corresponding to an uncorrected p < 0.05). As the statistical significance of these group differences had already been determined on the scalp level, the presence of some activation differences in underlying networks was considered to be statistically established. The role of sLORETA was thus not to determine statistical significance but to identify the most probable intracerebral generators of these differences in the amount (but not necessarily the distribution) of activation.

### Statistical Analysis of Clinical data

Between-group comparisons of socio-demographics and variables regarding hedonic experience, anhedonia, real-life motivation, and general cognitive abilities were performed with χ2 and one-way analysis of variance (ANOVA) tests, according to the variable type. For group comparisons on hedonic experience (TEPS scores), trait anhedonia (PAS and SAS), and quality of life, the scores of general cognitive abilities scores (WAIS-R) were used as a covariate in the analysis since differences between groups might have been influenced by this confounding factor.

To evaluate the relationships between the ERP-score with anticipatory hedonic experience and the two domains of negative symptoms, we performed partial correlation analyses using Pearson’s R correlation coefficient, adjusting for the confounding effects of general cognitive abilities. By correlating the ERP scores with the anticipatory TEPS scores and the negative symptoms evaluation, it is thus possible to draw conclusions about the relationship between group differences in the ERPs and the clinical evaluation.

Statistical Analyses were conducted with the Statistical Package for the Social Sciences (IBM SPSS Statistics), Version 25.

## Results

### Sample Characteristics

Three SCZ and two HC were excluded because they were unable to perform the target identification task during ERP recording (they achieved less than 60% of correct responses). Two SCZ and one HC were excluded for the presence of artifacts in the ERP recordings (less than 9 trials were available for averaging for the reward or loss conditions or less than 12 trials for the neutral one). Therefore, the analyses were conducted in thirty SCZ and twenty-three HC. Sociodemographic variables (i.e., age, gender, and years of education) were assessed in SCZ and HC groups and compared with one-way ANOVA or χ^2^ tests. SCZ and HC groups differed for years of education (p < 0.001). Furthermore, we observed a significant difference in general cognitive abilities (p < 0.001) and real-life motivation (p < 0.001) (Table 1). We observed no significant differences in anticipatory (p = 0.260) and consummatory (p = 0.956) hedonic experience, trait physical (p = 0.378), and social (p = 0.196) anhedonia between SCZ and HC, when controlling for general cognitive abilities.

**Table 1:** Demographic and clinical characteristics of the study sample

	SCZ (n = 30)	HC (n = 23)	F/χ^2^	p
Demographic information				
Age (mean ± SD)	34.23 ± 7.28	31.57 ± 8.18	1.57	0.216
Gender (M/F)	18/12	10/13	1.43	0.232
Education (mean ± SD)	12.33 ± 3.33	16.09 ± 2.71	19.39	**< 0.001**
General cognitive abilities				
WAIS-R Full-scale IQ	78.95 ± 15.69	112.67 ± 18.10	37.84	**< 0.001**
Real-life motivation, hedonic experience and anhedonia				
Real-life motivation	3.16 ± 1.46	5.85 ± 0.35	20.19	**< 0.001**∗
TEPS—anticipatory	3.98 ± 0.65	4.68 ± 0.43	5.41	0.261∗
TEPS—consummatory	4.07 ± 0.86	4.82 ± 0.61	0.0030	0.956∗
Trait physical anhedonia	24.32 ± 8.08	17.22 ± 6.56	0.80	0.378∗
Trait social anhedonia	14.68 ± 6.25	9.22 ± 3.89	1.74	0.196∗
Psychopathology				
PANSS positive	7.41 ± 4.35	–	–	–
PANSS negative	11.00 ± 6.68	–	–	–
PANSS disorganization	6.86 ± 3.43	–	–	–
SDS—Expressive deficit	2.68 ± 2.48	–	–	–
SDS—Motivational Deficit domain	4.23 ± 3.11	–	–	–

*SD* standard deviation, *SCZ* subjects with schizophrenia, *HC* healthy control, *PANSS* Positive and Negative Syndrome Scale, *SDz* Schedule for the Deficit Syndrome, *WAIS-R* Wechsler Adult Intelligence Scale-Revised, *TEPS*Temporal Experience of Pleasure Scale

p values in bold indicate statistical significance

∗Analyses were performed by entering general cognitive abilities (WAIS-R) as covariate

### ERP and Microstate Analysis

The topographic consistency test yielded, across the different conditions and groups, significant results in a time-range from 0 to 700 ms after cue presentation. We, therefore, restricted all following ERP analyses within this time window.

**Fig. 1:**
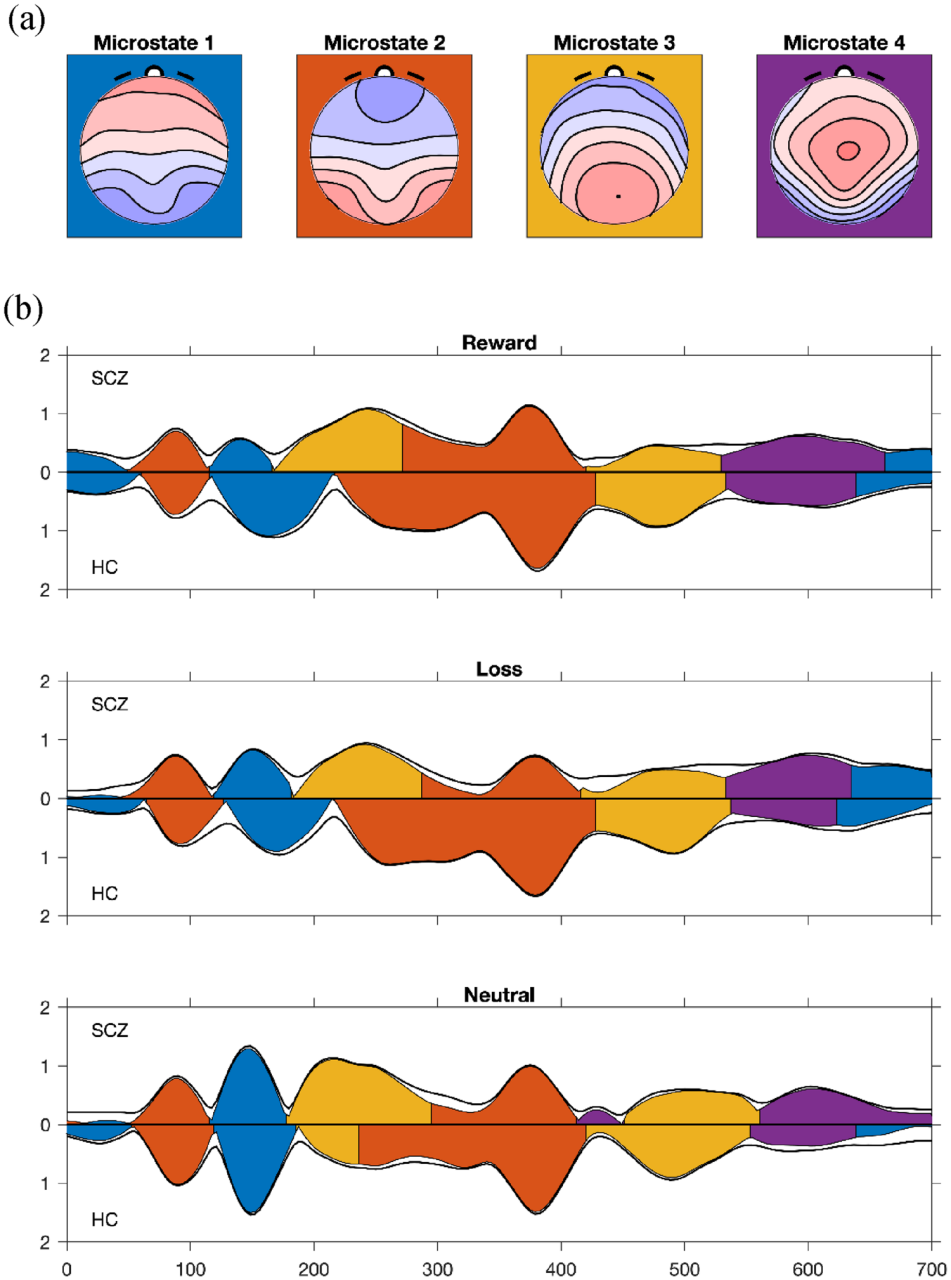
Microstate analysis. **a** 4 microstate maps computed for the ERPs of patients and controls across the three conditions (reward/loss/control); **b** microstate assignment to the ERPs of patients (SCZ) and controls (HC). The assignment of a microstate to a specific time point is indicated by color-coding depicted under the respective GFP curve. The y-axis indicates that the GFP curve of patients with schizophrenia is plotted with positive values up, while the healthy controls ERPs are flipped and plotted with positive values down. The x-axis represents time (ms) after cue stimulus presentation; the y-axis refers to the global field power, which is displayed in microvolts (µV)

When clustering the ERP for the microstate analysis and applying cross-validation to the number of microstate classes (Koenig et al. 2014), the percent explained variance in the test set ceased to increase after more than 6 classes. However, it can be observed that the six microstate classes solution (Fig. S2), displayed two final classes (classes 5 and 6), which occurred as very brief and low-GFP microstates, present mainly in transitioning time windows between the most prominent microstate classes. A closer inspection and comparison of the microstate models obtained with different numbers of classes showed that lowering the number of classes to four (Fig. 1) eliminated brief, late, and low-GFP microstates that were not considered of particular interest in the current analysis and that merely inflated the number of tests. We, therefore, chose to conduct the rest of our analysis with 4 microstate classes that explained 83.7% of the variance of the ERP in the given time window. The obtained maps of microstate classes and their distribution across time as a function of condition and group are shown in Fig. 1.

#### Analysis of Microstate Class 1 (MS1)

The first microstate class (MS1) appears to occur three times during the time window selected (Fig. 1). Analysis on a very early time window (time window considered: 0-100 ms after cue presentation) showed no significant group × stimulus interaction (p > 0.05). Analysis of the second appearance of MS1 (time window considered: 100–300 ms) revealed an interaction effect between group and condition for four of the six features considered. The offset (p = 0.00020), the duration of the microstate (p = 0.0026), the center of gravity (p = 0.0012) and the area under the curve (p = 0.0048) all showed a significant interaction effect between the group and cue type (condition) factors. Therefore, post-hoc tests were implemented to compare the differences between groups and conditions in these four parameters.

**Fig. 2:**
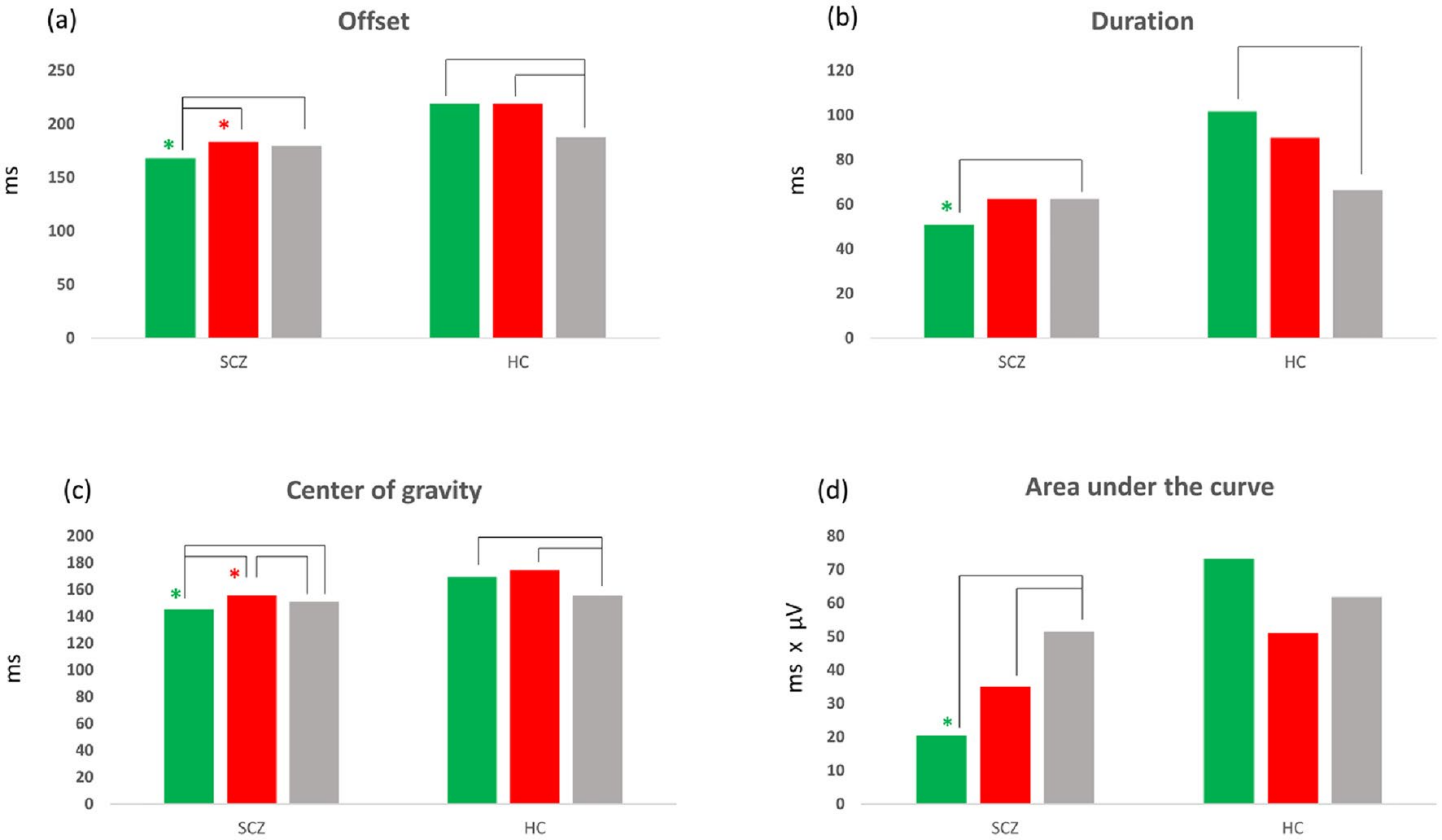
Bar graphs for MS1 features for the second occurrence of this microstate class. The bar graphs show differences in mean values for offset (**a**), duration (**b**), center of gravity (**c**), and area under the curve (**d**) of the MS1 for reward (green), loss (red), and neutral (gray) conditions in patients with schizophrenia (SCZ) and healthy controls (HC). Significant differences between conditions (p < 0.05) in each of the two groups were highlighted by black lines, while differences between groups in the same condition were flagged with the asterisk of the corresponding condition color

For the reward condition, the temporal features of the microstate all showed a similar inversion pattern when considering the two groups (Fig. 2). Specifically, in HC the offset (p = 0. 011) (Fig. 2a), the duration (p = 0.0036) (Fig. 2b) and the center of gravity (p = 0.0056) (Fig. 2c) values of the first microstate for the reward condition displayed a longer duration and a later ending as compared to the neutral condition. On the other side, SCZ displayed an opposite pattern in these same temporal features of the first microstate, since the offset (p = 0.0024), the duration (p = 0.035) and the center of gravity (p = 0.014) parameters indicated a shorter duration and earlier end in the reward condition as compared to the neutral one. Furthermore, analysis between groups showed that for the reward condition, SCZ had earlier offset (p = 0.0010), shorter duration (p = 0.020) and earlier center of gravity (p = 0.0010), as compared to the values recorded for the same condition in HC.

In addition to these temporal features, the area under the curve values showed that in HC, there was no statistically significant (p > 0.05) difference between the reward and neutral condition (although the mean values recorded were higher in the reward condition as compared to the neutral one). Conversely, SCZ, displayed significantly smaller values (p = 0.00020) (Fig. 2d) for the reward condition as compared to the neutral one for this parameter representing the magnitude of the microstate. Furthermore, the values in the reward condition were also significantly smaller (p = 0.019) in SCZ when compared to HC.

For the loss condition, the results showed some significant differences between groups, but the marked inversion of pattern considering the neutral condition noticed in the reward condition was not present. Specifically, in HC the offset (p = 0.042) and the center of gravity (p = 0.0002), but not the duration (p > 0.05), of the loss condition showed a delayed end as compared to the neutral condition and similar values to the reward one. Conversely, in SCZ, the offset (p > 0.05) and duration (p > 0.05) were not statistically different from the neutral condition, while the center of gravity was later in the loss as compared to the neutral condition (p = 0.022). When considering between-groups differences in the loss condition, the offset (p = 0.013) and center of gravity (p = 0.00060) were significantly different between the two groups (earlier offset and center of gravity in SCZ), while the duration did not change (although a trend difference was observable at the mean level). Furthermore, the area under the curve for the loss condition was significantly smaller than the neutral condition in SCZ (p = 0.039), but no significant difference in this feature in the loss condition emerged between the two groups (p > 0.05).

Post-hoc contrasts analysis showed that the neutral condition was not statistically different between the two groups for any of the features of the microstate considered (p > 0.05).

Finally, no significant group × type of stimulus interaction (p > 0.05) was recorded for the last occurrence of MS1 (time window considered: 600–700 ms).

#### Analysis of Microstate Class 2 (MS2)

For microstate class 2 (MS2), no significant interaction was present in the very early time window of its appearance (time window considered: 0-150 ms). Analysis of the second appearance of MS2 revealed an interaction effect between group and condition for two of the six microstate features considered (time window considered: 150–450 ms). The area under the curve (p = 0.0076) and the GFP (p = 0.015) showed a significant interaction effect between group and the condition variables. Therefore, post hoc tests were implemented to compare the differences between groups and conditions in these two parameters.

**Fig. 3:**
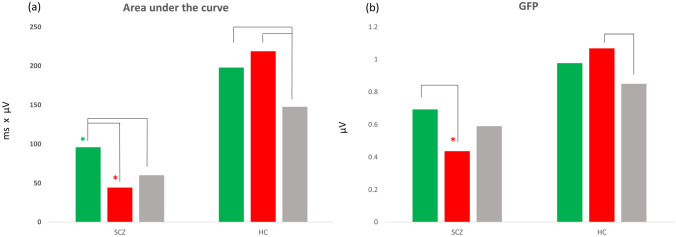
Bar graphs for MS 2 features for the second occurrence of this microstate class. The bar graphs show differences in mean values for the area under the curve (**a**) and GFP (**b**) of the MS 2 for reward (green), loss (red), and neutral (gray) conditions in patients with schizophrenia (SCZ) and healthy controls (HC). Significant differences between conditions (p < 0.05) in each of the two groups were highlighted by black lines, while differences between groups in the same condition were flagged with the asterisk of the corresponding condition color

For the reward condition, both HC (p = 0.024) and SCZ (p = 0.039) showed a bigger area under the curve as compared to the neutral one (Fig. 3a), while no differences between these two conditions were detected for GFP (Fig. 3b). Furthermore, in the reward condition, comparisons between groups showed that the area under the curve was significantly smaller (p = 0.031) in SCZ as compared to HC, while no significant between-groups difference (p > 0.05) was detected in the GFP.

For the loss condition, in HC, subjects showed significantly higher values (p = 0.022) for the area under the curve and the GFP (p = 0.048) for the loss condition as compared to the neutral one. In SCZ, no significant differences (p > 0.05) were detected between the loss and neutral conditions for the area under curve and GFP parameters. Furthermore, in the loss condition, comparisons between groups showed that the area under the curve was significantly smaller (p = 0.0008) and the mean GFP smaller (p = 0.0050) in SCZ as compared to HC. Post-hoc contrasts analysis showed that the neutral condition was not statistically different between the two groups for any of the features of the microstate considered (p > 0.05).

#### Analysis of Microstate Class 3 (MS3)

No significant group × condition interaction (p > 0.05) appeared in the second microstate in any of the features considered (time window considered: 150–350 ms). However, a main effect of group in this time window was detected for the onset (p = 0.031), offset (p = 0.041), duration (p = 0.020), area under the curve (p = 0.010) and mean GFP (p = 0.020) parameters, since in the HC group the MS3 did occur in the reward and loss condition in this time window, as it can be seen for the SCZ group. Finally, no significant interaction was present in the second occurrence of MS3 (time window considered: 400–600 ms).

#### Analysis of Microstate Class 4 (MS4)

No significant group × condition interaction (p > 0.05) appeared in the fourth microstate class in any of the features considered (time window considered: 500–700 ms).

### Correlation Analysis

We performed correlations between the ERP scores and anticipatory TEPS in the whole sample for both microstates of interest (MS1 and MS2) and the respective conditions of interest (reward for MS1 and loss for MS2).

**Fig. 4:**
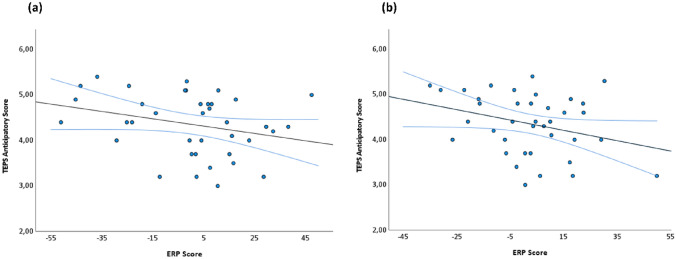
The two scatter-plots illustrate the significant correlation between ERPs scores recorded in the time windows of MS1 (reward condition) (r = − 0.295; 95% C.I. [− 0.486; 0.009]) (**a**) and of the MS2 (loss condition) (r = − 0.412; 95% C.I [− 0.430; 0.059]) (**b**) and the TEPS anticipatory scores. Specifically, when a subject presented a low (negative) ERP score, this indicated that the microstate map explained less of the ERP data of that subject in comparison to the mean of all subjects in the given group and that, therefore, the brain functions associated with this microstate map were less active compared to the mean of the group. Therefore, the negative correlation observed in both plots suggests that subjects with a low ERP score also had greater difficulties anticipating pleasure (higher TEPS). The black lines represent the lines of best fit of the correlation, while the light blue ones indicate the confidence interval (95%) of the correlation

The analysis showed that the ERP scores of both reward in MS1 (125–187.5 ms) (Fig. 4a) and loss in MS2 (261.7–414.1 ms) were significantly correlated with the anticipation of pleasure scores. Specifically, a significant negative correlation was recorded for both MS1-reward (r = − 0.295; p = 0.041; 95% C.I. [− 0.486; 0.009]) (Fig. 4a) and MS2-loss (r = − 0.412; p = 0.006; 95% C.I [− 0.430; 0.059]) (Fig. 4b).

Finally, in SCZ, we performed a correlation between ERP scores of the same microstate time windows and conditions with the severity of the two negative symptom domains; however, no significant correlations were found (p > 0.05).

### Source Analysis

sLORETA source analysis was performed in order to investigate the brain regions generating significant main effects and interactions regarding GFP. This analysis considered only the time frame and condition that reported a significant group effect in the microstate analysis (onset and offset of the grand average of the microstate across conditions and groups), as we took this as sufficient evidence for differences in active sources.

#### Second Time Window of MS1– Reward Condition

**Fig. 5:**
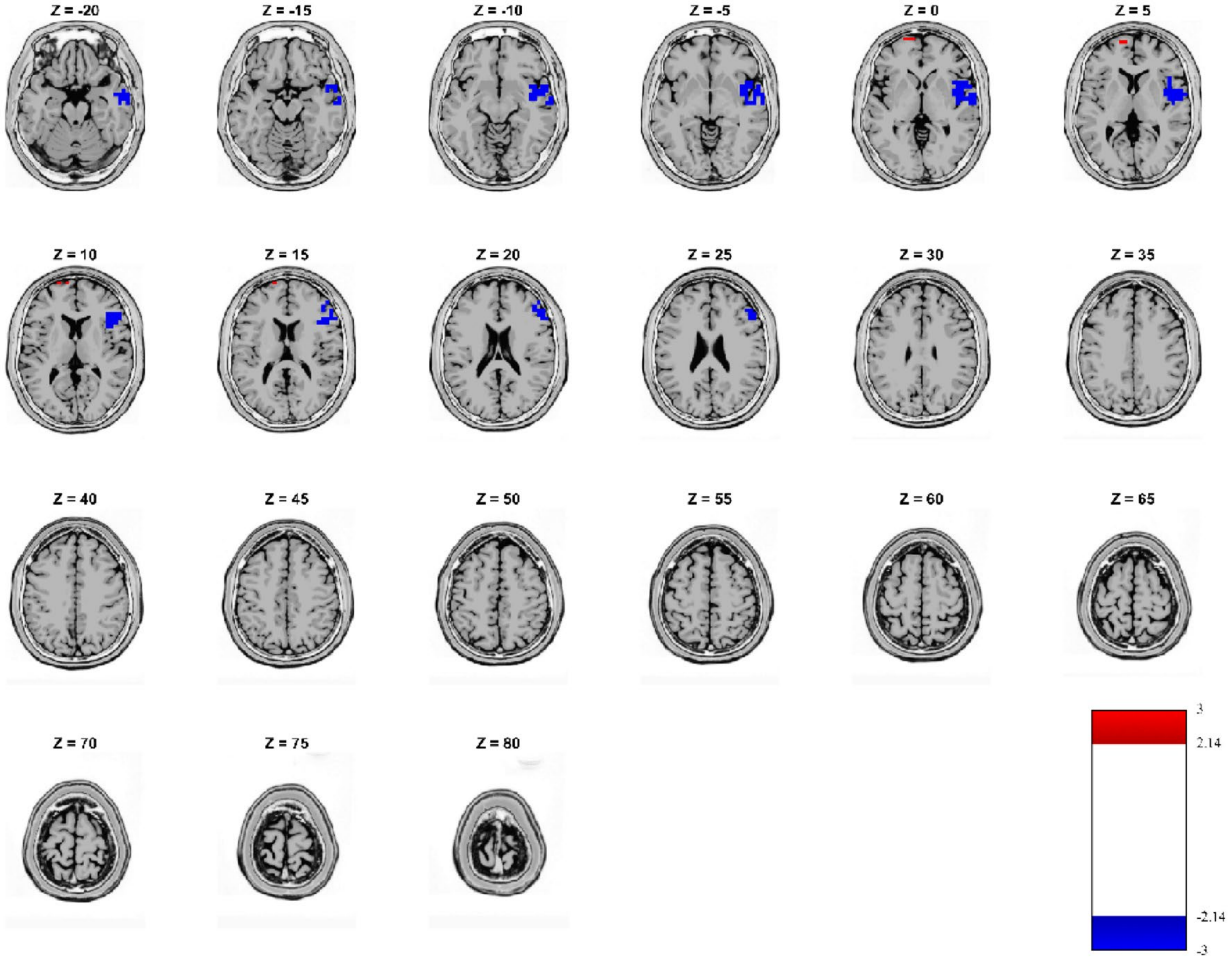
sLORETA localizations. Source estimations of significant effects (voxel-wise *t* test thresholded at − 2.14 and 2.14; p < 0.05 uncorrected) in the analyses of subjects with schizophrenia and controls for the second occurrence of MS1 time window in reward condition. Blue regions indicate a significant lower activity in SCZ as compared to HC, while red regions flag higher activity in SCZ vs. HC

The source analysis compared SCZ and HC for the first microstate for the reward condition considering the significant 125.0 and 187.5 ms time window (Fig. 5). In SCZ, hypo-activation was detected in the insula, superior temporal gyrus and orbitofrontal cortex of the right hemisphere as compared to HC. In addition, a very small region of increased activity in SCZ as compared to HC was recorded in the left prefrontal cortex and medial frontal gyrus (Fig. 5).

#### Second time Window of MS2—Loss Condition

**Fig. 6:**
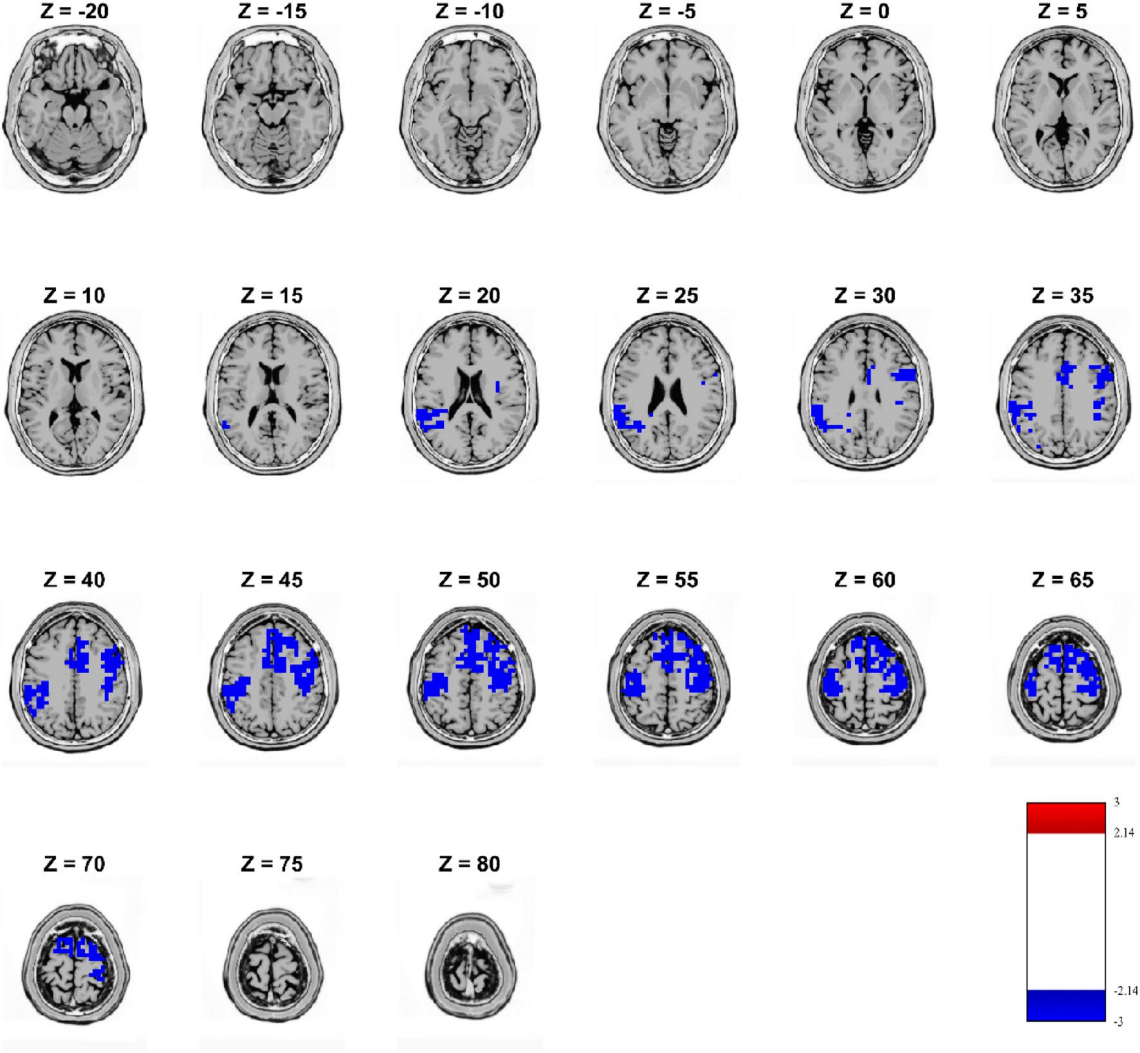
sLORETA localizations. Source estimations of significant effects (voxel-wise *t* test thresholded at − 2.14 and 2.14; p < 0.05 uncorrected) in the analyses of subjects with schizophrenia and controls for the second occurrence of MS2 time window in the loss condition. Blue regions indicate a significantly lower activity in SCZ as compared to HC

Source analysis of the significant time window of the second microstate (261.7–414.1 ms) revealed that in loss condition, differences in source activity were recorded as a lower activity in SCZ as compared to HC (Fig. 6). Specifically, these regions of hypoactivity were recorded bilaterally in the superior frontal gyrus, motor cortex and middle frontal gyrus. Furthermore, the left hemisphere presented lower activity in SCZ as compared to HC in the voxels localized in the parietal and temporal lobules, while in the right hemisphere specific significant hypoactivity was recorded in the cingulate cortex, in the insula and parts of the somatosensory cortex (Fig. 6).

## Discussion

The present study had three primary objectives: (1) to investigate electrophysiological differences in microstates configuration between individuals with schizophrenia and healthy controls during the anticipatory phase of the MID task; (2) to analyze the associations between ERP scores during the MID-task-cue stage and the scores for anticipatory pleasure and negative symptoms; (3) to explore variations at the source level for ERPs during reward anticipation and loss avoidance stages.

The first outcome of the present study showed that differences between patients and controls were already detectable in the configuration of an early microstate component (MS1). As demonstrated by our results and according to those presented by Flores and colleagues, it is possible that the type of cue presented, and its valence to the subjects can influence the characteristics of very early ERPs (Flores et al. 2015). Differences in responses to the presentation of a specific cue (reward, loss, neutral) have indeed been detected 100–200 ms post-stimulus, as suggested by increases in N100 and P200 amplitude when reward cues were presented as compared to neutral ones (Yu and Zhou 2006; Doñamayor et al. 2012), suggesting that subjects allocated greater attentional resources towards stimuli flagging incoming rewards. The interpretation of our results fits within this frame. In particular, we observed that, in healthy controls, some parameters of the earlier microstate differed between the reward and the neutral conditions. Specifically, the first microstate showed a longer duration and a later offset for the reward condition. This pattern was reversed in subjects with schizophrenia, since a shorter duration and earlier end of the first microstate in the reward condition was recorded when compared to the neutral one. These results suggest a reduced engagement of neuronal resources devoted to the processing of cue related to incoming rewards in patients, which could indicate a reduced allocation of attention to stimuli anticipating pleasant events.

Furthermore, we found a correlation between the ERP scores, calculated within the time window of the second presentation of MS1, and anticipatory hedonic experience scores in the whole group. This supports the hypothesis that the characteristics of MS1 might be associated with the efficiency in processing incoming rewards. No correlations were found, instead, with negative symptoms in the patient group. The number of studies focusing on the associations between early ERPs, such as the N100, and the severity of negative symptoms is low, and the outcomes have not been univocal: some studies found significant associations between N100 and negative symptoms (Giordano et al. 2021a; Sumich et al. 2006; Mucci et al. 2007), while one study did not (Li et al. 2013). Furthermore, it is difficult to interpret these results in the context of reward processing since these studies have mainly used an auditory paradigm during the EEG recordings, which was not related to anticipation of pleasant events as in the MID task.

With regard to the loss-avoidance condition, our results demonstrated that MS1 had a delayed end in loss as compared to the neutral condition in healthy controls. At the same time, subjects with schizophrenia expressed no differences between these conditions, thus suggesting a pathological flattening of the processing of loss anticipation.

Overall, the results regarding the MS1 indicate that alterations in schizophrenia can be recorded already during early stages of reward processing and might be linked to impaired anticipation of pleasure, as suggested by alterations in the microstates parameters and by the association between the ERPs scores and the scale evaluating anticipation of hedonic experiences.

Beyond these very early neurophysiological alterations, differences between subjects with schizophrenia and healthy controls were also detectable in the second microstate class, occurring between 200 and 400 ms after the presentation of the cue. It is of interest to note that, although no previous ERP studies using the MID task focused on the early time window, different studies investigated later ERPs both in healthy controls and patients with schizophrenia (Potts 2011; Novak and Foti 2015; Pornpattananangkul and Nusslock 2015; Dunning and Hajcak 2007; Osinsky et al. 2014; Vignapiano et al. 2016, 2018; Polich and Kok 1995; Hughes et al. 2013:Broyd et al. 2012b; Gruber and Otten 2010). It was shown that, in healthy controls, the amplitude of the ERP N200 (elicited approximately 200–300 ms after cue presentation) was increased for cues flagging potential loss compared to neutral cues (Glazer et al. 2018; Vignapiano et al. 2018),which might flag a process mediated by cognitive control to avoid possible future losses (Glazer et al. 2018; Potts 2011; Novak and Foti 2015), a feature that seems to be impaired in schizophrenia (Carpenter 2021; Vignapiano et al. 2018). In addition to N200, the elicitation of P300 has been extensively studied in the context of reward processing (Polich and Kok 1995; Geal-Dor et al. 2006; Polich 2007; Hughes et al. 2013; Vignapiano et al. 2016; Glazer et al. 2018). Both reward and punishment anticipation elicit greater P300 amplitudes than the neutral condition and the enhancement of this ERP seems to reflect the stimulus-categorization processes, which are primarily involved in motivated attention for obtaining rewards and avoiding losses (Geal-Dor et al. 2006; Pornpattananangkul and Nusslock 2015; Novak and Foti 2015; Glazer et al. 2018; Broyd et al. 2012a; Pfabigan et al. 2014).

In line with these results, our findings indicate that in healthy controls, some parameters of the second appearance of the MS2 (time window: 150–450 ms) were larger for reward (area under the curve) and loss (area under the curve and the global field power) conditions as compared to the neutral one. Conversely, in patients with schizophrenia, the enhancement in neuronal responses resulted attenuated after the presentation of the reward cues, and disappeared when the loss cues were considered, suggesting impairments in processing also during later stages.

As for the first microstate class, the ERP scores of the MS2, were also associated with the anticipation of hedonic experience in the whole group. No correlations were found with negative symptoms in the patient group, confirming previous studies that did not report a relationship between late ERPs (N200 and P300) and negative symptoms in treated patients with schizophrenia (Vignapiano et al. 2016; Mucci et al. 2007; Giordano et al. 2021b; Jeon and Polich 2003). Therefore, the outcomes of these correlations suggest that that negative symptoms have multiple pathophysiological mechanisms and that deficits in motivation and anticipation of pleasant experiences are partially independent constructs (Vignapiano et al. 2016).

Finally, another result that was highlighted was the presence of a main effect of group for the first occurrence of MS3. However, the fact that no interaction effect was detected between the group and condition, suggests that this effect might not be strictly related to the task employed or to reward processing.

Overall, the findings on MS2 are in accordance with the studies reporting how reward processing influence the elicitation of ERPs occurring in the same time window, such as the N200 and P300, during the MID task.

To analyze the electrophysiological differences recorded in microstate features at the source-level, sLORETA analysis was implemented as a follow-up investigation. Our analysis revealed that subjects with schizophrenia showed dysfunctions in areas belonging to the motivational circuits, as demonstrated by alterations in the activity of the orbitofrontal cortex (OFC), cingulate cortex, prefrontal cortex, superior temporal gyrus (STG), parietal areas and insular cortex, as compared to healthy controls (Galderisi et al. 2018; Amodio et al. 2018; Giordano et al. 2021c; Zeng et al. 2022; Dumas 2022). Different alterations in brain activity and connectivity within these areas have already been reported in subjects with schizophrenia (Bègue et al. 2020; Galderisi et al. 2018; Krueger et al. 2021; Kirschner et al. 2016; First et al. 2021; Lahey et al. 2021; Mucci et al. 2015, 2019a).

In our study, the insular cortex showed a decreased activation in subjects with schizophrenia, as compared to healthy controls. Previous studies showed that the insular cortex is an important interface node between the Motivational Value and the Motivational salience systems, providing a link between processing of external information and internal motivational states(Galderisi et al. 2018; Harsay et al. 2012; Chikama et al. 1997; Wilson et al. 2018; Watson et al. 2022).

A decrease in activity in subjects with schizophrenia was also found for the cingulate cortex, which has a key role in integrating cognitive control and motivational processes for goal-directed behavior (Cadena et al. 2018; Ballard et al. 2011; Fornito et al. 2009; Giordano et al. 2018a, 2022a; Carpenter 2021; Lysaker and Hasson-Ohayon 2021), as also confirmed by a meta-analysis focusing on the MID task paradigm (Wilson et al. 2018). Therefore, alterations within this area, might lead to impairments in cognitive control, general motivation, and the ability to efficiently devote attentive resources towards salient stimuli; aspects which are often compromised in schizophrenia and of primary importance in general in the management of mental health (Zeng et al. 2022; Ventura 2022; Borsboom et al. 2022; Patton et al. 2021).

Both the insular and cingulate cortices have also extensive connections with other brain regions involved in reward-related processes including the OFC and the STG, which both showed alterations in our sample of patients. These areas are thought to be involved in encoding reward outcomes and determining the effort required to obtain them.

Therefore, the differences in the activity levels of these regions might be interpreted as a neurobiological marker of deficits in making efficient predictions about future outcomes, which would guide reward-based decisions (Morrison et al. 2011; O’Doherty 2007, 2011; Bissonette and Roesch 2016; Reddy et al. 2016; Waltz and Gold 2016; Waltz et al. 2013; Steinberg et al. 2013; Holt et al. 2009; Murray et al. 2008; Gold et al. 2008; Stein et al. 2022; Mucci et al. 2015; Rolls et al., 2019; Rolls et al. 2020; Rushworth et al. 2012). Finally, our results are also in line with a meta-analysis focused on fMRI studies in subjects with schizophrenia employing the MID task, which revealed that during the anticipatory phase of the task, patients exhibited hypo-activation in the insula, anterior cingulate cortex and superior temporal gyrus (Zeng et al. 2022).

However, despite the interesting insights given by the current study, certain limitations should be taken into account. First, the sample size of the current study is limited. Second, the cut-off values for the band-pass filter during EEG data preprocessing were different from other studies focusing on the MID task (Flores et al. 2015; Novak and Foti 2015), which should be considered for future comparisons with the present research. Finally, the sLORETA results are constrained by the limited spatial resolution and inevitably cannot be considered as precise as fMRI findings.

In conclusion, the outcomes of the current study show that in subjects with schizophrenia, abnormalities in ERPs may be detected both during the early stages of reward processing and at later stages in the anticipation of loss-avoidance.

These results suggest the presence of different impairments in the effective evaluation of incoming experiences in subjects with schizophrenia. The present study highlights that electrophysiological abnormalities might be present even during early stages of reward processing. Therefore, further studies aimed at investigating the pathophysiological bases of these deficits, even with more sophisticated analysis techniques, such as machine learning analysis (Chekroud et al. 2021), combining the use of multiple neuroimaging indices simultaneously (early and late ERPs), are strongly encouraged to promote knowledge in this field.

### Supplementary Information

Below is the link to the electronic supplementary material.

Supplementary material 1 (DOCX 865.6 kb)

## Data Availability

The datasets generated during and/or analyzed during the current study are available from the corresponding author upon reasonable request.
