# Supplementary material for: Electrophysiological Correlates of Reward Anticipation in Subjects with Schizophrenia: An ERP Microstate Study
Source: Brain Topogr. 2023 Jul 5;37(4):1–19. doi: 10.1007/s10548-023-00984-7 (PMC11199294; doi:10.1007/s10548-023-00984-7)
Supplement: Supplementary file 1 — Supplementary material 1 (DOCX 865.6 kb) [file 10548_2023_984_MOESM1_ESM.docx]

**Supplementary material**

Electrophysiological correlates of reward anticipation in subjects with schizophrenia: an ERP microstate study

**Authors**: Perrottelli, A.^,^ Giordano, G.M., Koenig, T.*, Caporusso, E, Giuliani, L., Pezzella, P., Bucci, P., Mucci, A., Galderisi, S.

***Corresponding author**: Professor Thomas Koenig, University Hospital of Psychiatry and Psychotherapy, Translational Research Center, University of Bern, Switzerland; thomas.koenig@upd.unibe.ch

**Journal:** Brain Topography

**
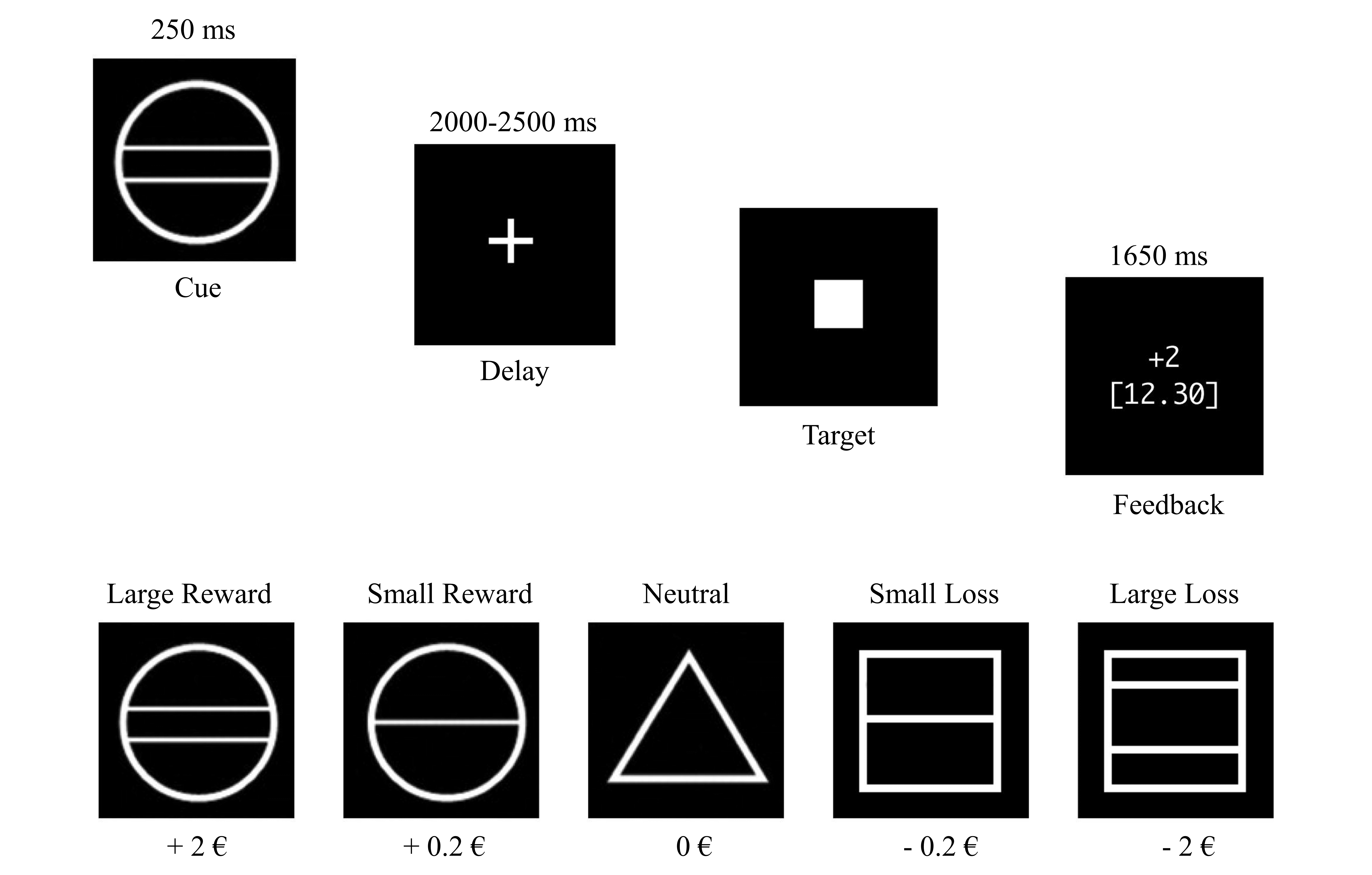
Supplementary Figures:**

**Fig. S1.** Monetary Incentive Delay task. During each trial, the subject randomly saw one of 5 different cues (shown at the bottom) for 250 ms (in this case, the circle with 2 lines is the cue anticipating a large reward), then fixated a crosshair during a variable anticipation interval (“delay”: 2000–2500 ms), and pressed a button when a white square (target) was presented. An outcome feedback (1650 ms) followed the target presentation, informing the subject on the amount of money gained (2€ in this case) or lost in that trial, as well as on the cumulative outcome across trials (12.30€ for the entire experiment).

**
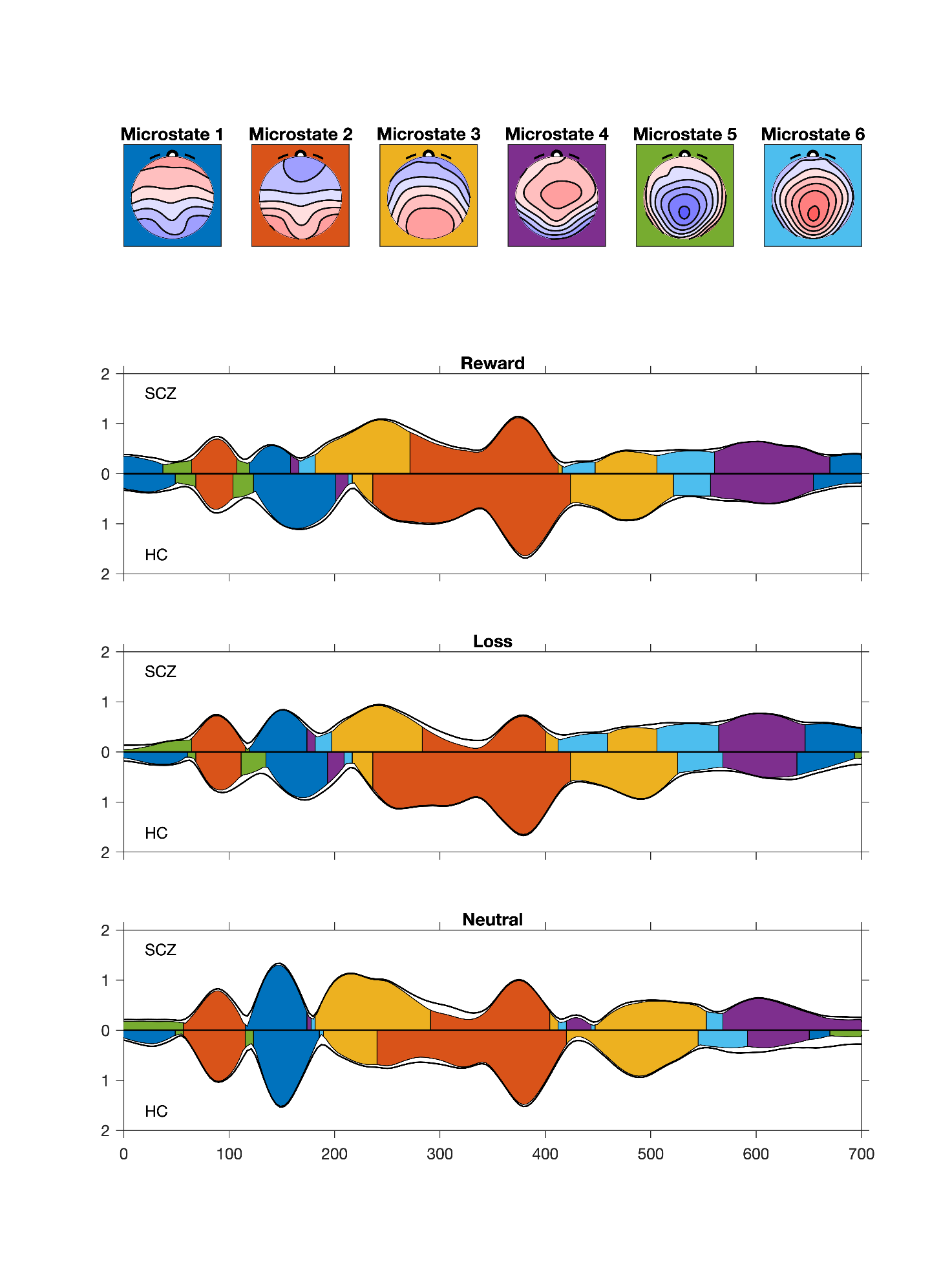
**

(a)

(b)

**Fig. S2.** Microstate analysis with 6 classes. (a) 6 microstate maps computed for the ERPs of patients and controls across the three conditions (reward/loss/control); (b) Microstate assignment to the ERPs of patients (SCZ) and controls (HC). The assignment of a microstate to a specific time point is indicated by color-coding depicted under the respective GFP curve. The y-axis indicates that the GFP curve of patients with schizophrenia is plotted with positive values up, while the healthy controls ERPs are flipped and plotted with positive values down. The x-axis represents time (ms) after cue stimulus presentation; the y-axis refers to the global field power, which is displayed in microvolts (µV).
